# Large herbivores maintain a two‐phase herbaceous vegetation mosaic in a semi‐arid savanna

**DOI:** 10.1002/ece3.5750

**Published:** 2019-10-22

**Authors:** David J. Augustine, Benjamin J. Wigley, Jayashree Ratnam, Staline Kibet, Moses Nyangito, Mahesh Sankaran

**Affiliations:** ^1^ Rangeland Resources and Systems Research Unit United States Department of Agriculture – Agricultural Research Service Fort Collins CO USA; ^2^ National Centre for Biological Sciences Tata Institute of Fundamental Research Bangalore India; ^3^ School of Natural Resource Management Nelson Mandela University, George Campus George South Africa; ^4^ Department of Resource Management and Agricultural Technology University of Nairobi Nairobi Kenya; ^5^ School of Biology Faculty of Biological Sciences University of Leeds Leeds UK

**Keywords:** alternative stable states, equilibrium versus nonequilibrium dynamics, grazing management, reversible degradation, vegetation collapse, vegetation patch dynamics

## Abstract

1. Many arid and semi‐arid rangelands exhibit distinct spatial patterning of vegetated and bare soil‐dominated patches. The latter potentially represent a grazing‐induced, degraded ecosystem state, but could also arise via mechanisms related to feedbacks between vegetation cover and soil moisture availability that are unrelated to grazing. The degree to which grazing contributes to the formation or maintenance of degraded patches has been widely discussed and modeled, but empirical studies of the role of grazing in their formation, persistence, and reversibility are limited.
2. We report on a long‐term (17 years) grazing removal experiment in a semi‐arid savanna where vegetated patches composed of perennial grasses were interspersed within large (>10 m^2^) patches of bare soil.
3. Short‐term (3 years) grazing removal did not allow bare patches to become revegetated, whereas following long‐term (17 years) grazing removal, bare soil patches were revegetated by a combination of stoloniferous grasses and tufted bunchgrasses. In the presence of grazers, stoloniferous grasses partially recolonized bare patches, but this did not lead to full recovery or to the establishment of tufted bunchgrasses.
4. These results show that grazers alter both the balance between bare and vegetated patches, as well as the types of grasses dominating both patch types in this semiarid savanna.
5. Synthesis: Large herbivores fundamentally shaped the composition and spatial pattern of the herbaceous layer by maintaining a two‐phase herbaceous mosaic. However, bare patches within this mosaic can recover given herbivore removal over sufficiently long time scales, and hence do not represent a permanently degraded ecosystem state.

## Graphical abstract

The stability and spatial pattern of degraded and vegetated patches in dryland ecosystems has been modeled and discussed for decades, with few empirical studies. We present one of the first long‐term experiments that demonstrate these patterns are maintained by large herbivore grazing and can disappear when herbivores are removed.

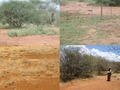

## 1 INTRODUCTION

The degree to which domestic and wild large herbivores influence vegetation dynamics in rangelands can vary widely, and ecosystem‐ or landscape‐scale factors influencing vegetation resilience to grazing have been the subject of considerable research and debate for the past several decades (Anderson, Ritchie, & McNaughton, 2007; Augustine & McNaughton, 1998; Ellis & Swift, 1988; Illius & O'Connor, 1999; Milchunas & Lauenroth, 1993; Oba, Stenseth, & Lusigi, 2000). The potential for grazing‐induced loss of vegetation to result in a largely irreversible and degraded state dominated by bare soil and/or sparse annual grasses has been widely discussed and modeled (e.g., Bestelmeyer, 2006; King & Franz, 2016; Le Houerou, 1986; Milton, Dean, Plessis, & Siegfried, 1994; Van de Koppel et al., 2002, 1997), and has major implications for the long‐term productivity of rangelands in Africa and worldwide. The concepts of thresholds and irreversible change in rangeland condition are closely related to the scale and pattern of vegetation cover in these ecosystems (Van de Koppel et al., 2002). Many arid and semi‐arid rangelands exhibit distinct spatial patterning of bare soil‐dominated versus vegetated patches (Aguiar & Sala, 1999; Ludwig, Wilcox, Breshears, Tongway, & Imeson, 2005), and the size and connectivity of such patches have been proposed as one means to identify thresholds in rangeland condition beyond which irreversible shifts in ecosystem states occur (Kefi et al., 2007, 2010). However, empirical studies of the role of grazing in the formation, persistence, and reversibility of bare soil patches are limited. Some long‐term grazing studies have demonstrated that rangelands on certain soil types with high water infiltration rates and minimal precipitation runoff can be resilient to a wide range of grazing intensities (e.g., Veblen, Porensky, Riginos, & Young, 2016), while others show that vegetation responses are contingent on stocking rates (e.g., Cipriotti & Aguiar, 2005; Fynn & O'Connor, 2000). Furthermore, certain soil types may be particularly sensitive to the formation of bare soil‐dominated patches where vegetation reestablishment is impeded, even after a reduction in grazing intensity (e.g., Franz et al., 2012; Kinyua, McGeoch, Georgiadis, & Young, 2010). On these types of soils, implementation of cost‐effective restoration strategies depends on understanding the conditions and time scales over which grazing management or removal could potentially restore vegetation cover (e.g., Kimiti, Hodge, Herrick, Beh, & Abbott, 2017).

Here, we report on a 17‐year herbivore exclusion experiment conducted in a semi‐arid Kenyan savanna characterized by a distinct, two‐phase mosaic of bare soil patches interspersed with vegetated patches. The latter are dominated by a diverse community of perennial grasses. Bare soil‐dominated patches are on the order of ~5–15 m in diameter, and in some cases are sufficiently large and interconnected so as to form a background matrix within which the vegetated patches are embedded (Augustine, 2003). These “bare soil” patches still contain some limited vegetation cover, which often consists of thin‐leaved, unpalatable grasses such as *Harpachne schimperii*, *Aristida* spp, and *Eragrostis tenuifolia* (Augustine, 2003), which have low‐forage value for grazing ungulates (PANESA, 1988; Stewart & Stewart, 2015). Previous short‐term experiments found that grazing by large herbivores influenced vegetation productivity, but not the composition, size or location of patches over time scales of 1–2 years (Augustine & McNaughton, 2006). The study area has been managed as a commercial ranch supporting moderate cattle densities for the past several decades, and surveys in the 1990s estimated that approximately one‐third of the landscape underlain by sandy soils is composed of bare soil‐dominated patches that produce almost no useable forage for livestock or native grazers (Augustine, 2003). These patches may represent areas that could support productive perennial grasses, but have been driven to an alternate stable state by grazing and the consequent loss of plant cover, organic inputs to the soil, and water infiltration capabilities (Rietkerk & van de Koppel, 1997). Alternatively, spatial feedbacks between soil moisture and plant growth may be the primary factor leading to the creation and maintenance of such vegetation patterns (Aguiar & Sala, 1999; Deblauwe, Barbier, Couteron, Lejeune, & Bogaert, 2008). These two hypotheses are not completely exclusive, and disentangling the relative contribution of herbivory versus soil‐water‐plant feedbacks in structuring dryland plant communities is a daunting task that will likely rely on integration of empirical research and modeling (King & Franz, 2016). To support such an effort, we use a long‐term herbivore exclusion experiment to test whether and over what temporal scale grazing removal influences this two‐phase mosaic of bare soil‐dominated patches interspersed with patches of perennial herbaceous vegetation.

## 2 MATERIALS AND METHODS

**Figure 1:**
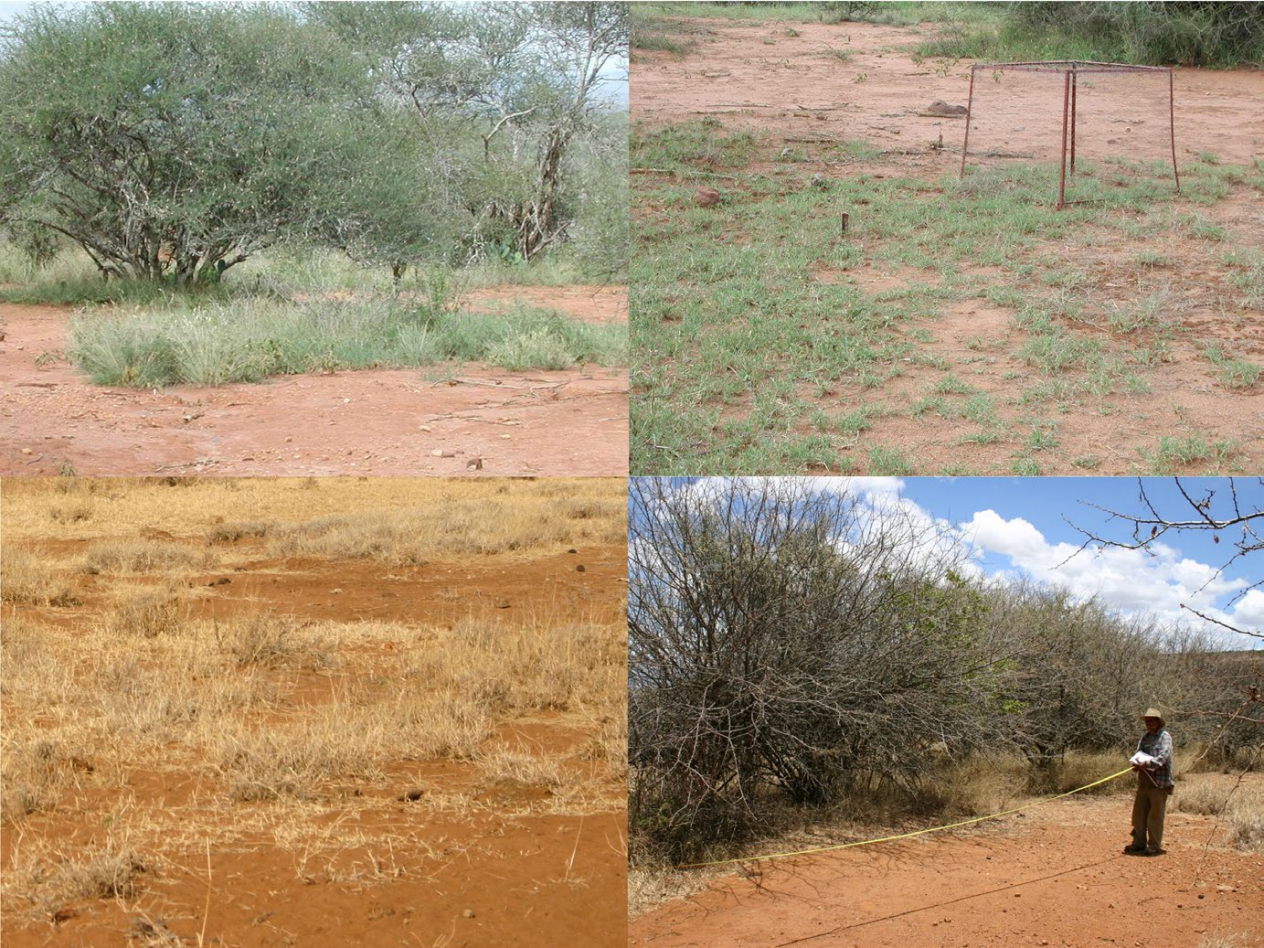
Examples of the two‐phase mosaic of bare and vegetated patches found in savannas underlain by sandy loam soils in Laikipia County, Kenya

The Mpala Conservancy (MC) consists of approximately 50,000 ha of semi‐arid rangeland managed for the production of commercial cattle which coexist with a diverse community of native large herbivores. Our study was conducted on the portion of MC underlain by deep, well‐drained, friable sandy loam soils developed from metamorphic basement material (Ahn & Geiger, 1987). Soils sampled throughout the study area (0–20 cm depth) average 76% sand, 1.1% carbon (C), and 0.1% nitrogen (Augustine, 2003). The topography consists of gently rolling hills. Mean annual rainfall increases from north to south, with a long‐term mean of 508 mm based on a rain gauge maintained near the center of our study area. The vegetation consists of a discontinuous mosaic of woody plants (~28%–33% cover) dominated by *Acacia etbaica*, *Acacia mellifera*, and *Acacia brevispica*, and a discontinuous understory herbaceous layer dominated by perennial C_4_ grasses, primarily *Digitaria milanjiana*, *Cynodon dactylon*, *Pennisetum mezianum*, and *Pennisetum stramineum* (Augustine, 2003; Augustine & McNaughton, 2006). Vegetated patches can include areas with a combination of a woody canopy and an herbaceous understory or an herbaceous layer lacking woody cover; the bare patches not only lack herbaceous cover, but also typically lack any woody overstory cover (Augustine, 2003; Figure 1). Previously, fire may have been an important driver of vegetation dynamics and herbivore distribution, but has been actively suppressed by ranch managers since European settlement. Over the past two decades, approximately 1,400–3,100 cattle have been maintained at Mpala, averaging 7.1 km^−2^. Native ungulate grazers consist of plains zebra (*Equus burchellii*), Grevy's zebra (*Equus grevyi*), waterbuck (*Kobus ellipsiprymnus*), and buffalo (*Syncerus caffer*), all of which occur at densities <1.5 km^−2^ (Augustine, 2010). The three most abundant native mixed‐feeding and browsing ungulates are impala (*Aepyceros melampus*; 20 km^−2^), dik‐dik (*Madoqua guentheri*; 70–139 km^−2^), and elephant (*Loxodonta africana*; 1.7 km^−2^; Augustine, 2010; Ford et al., 2015).

When we initiated this experiment in 1999, the herbaceous layer contained a mosaic of bare soil‐dominated patches on the order of ~5–15 m in diameter, which in some cases had become sufficiently large and interconnected to form a background matrix within which the vegetated patches were embedded (Augustine, 2003; Figure 1). We established the experiment at 3 replicate sites that were representative of this two‐phase mosaic. At each site, we demarcated two paired 50 × 50 m plots that were as similar to one another as possible in terms of herbaceous and woody plant composition and patch structure. We randomly selected one plot to be protected with a 70 × 70 m 11‐strand electrified exclosure that prevented access by all mammalian herbivores the size of a dik‐dik or larger, and one to serve as a control.

### 2.1 Measurements of herbaceous cover

Within each 50 × 50 m plot, we established a systematic grid of survey points (6 × 6 point grid), with points spaced at 10‐m intervals. We marked each point with a 1‐cm diameter iron rod driven into the ground. At each point, we measured cover of six plant functional groups in the herbaceous layer by placing a 1.1‐m long pin frame in each cardinal direction radiating from the survey point, and using the frame to insert 10 pins (spaced at 10 cm intervals) through the herbaceous layer at a 45‐degree angle. We recorded and analyzed data by plant functional groups to minimize errors associated with identifying some of the plants to the species level (particularly when they were grazed), while still differentiating among groups of grasses with notably different growth forms and differences in forage value for large herbivores.

The six functional groups for which we measured aboveground, live plant cover were as follows: (a) stoloniferious perennial grasses (*C. dactylon*, *Cynodon plectostachyus*, and *D. milanjiana*), (b) tufted perennial bunchgrasses (wide‐leaved, caespitose, perennial bunchgrasses with leaves primarily growing from basal tillers; *Themeda triandra*, *Enteropogon macrostachyus*, *Eragrostis superba*, *Heteropogon contortus*, and *Cymbopogon pospischilii*), (c) ascending perennial bunchgrasses (perennial grasses with leaves growing from vertically oriented stems; *P. stramineum*, *P. mezianum*, *Cenchrus ciliaris*, *Bothriochloa insculpta*, *Chloris* spp.), (d) low‐forage‐value graminoids (annuals and thin‐leaved, caespitose, perennial graminoids: *H. schimperii*, *Aristida* spp., *E. tenuifolia*, *Sporobolus festivus*, *Cyperus* spp., and *Michrochloa kunthii*), (e) forbs (highly diverse, including species in the genera *Commelina*, *Indigofera*, *Ipomoea*, *Oxygonum*, *Portulaca*, and *Ruellia*), and (f) dwarf shrubs (diverse, including *Solanum incanum*, *Barleria* spp., *Hibiscus* spp., *Ipomoea spathulata*, *Justicia diclopteroides*, *Melhania velutina*, *Oscimum* sp., *Plectranthes* spp, and *Phyllanthus suffructescens*). The stoloniferious grasses (STG) spread vegetatively and often assume a prostrate, lawn‐like growth form when grazed. In contrast, the tufted and ascending bunchgrasses rely primarily on seedling recruitment for long‐term persistence. Leaves of tufted bunchgrasses (TBG) primarily grow from basal meristems, and in the absence of reproductive culms, their growth form facilitates grazers removing bites with a large proportion of leaf material. Leaves of ascending bunchgrasses (ABG) often grow from vertically oriented stems, leading grazers to consume a combination of leaf and stem material when grazing this functional group. The low‐forage‐value graminoid (LFVG) functional group consists of a combination of perennial and annual species that either have thin, tough leaves with low digestibility, and/or thin, prostrate leaves that minimize intake by large herbivores. For each pin in the frame, we recorded whether or not it made contact with any live plant material in each of the 6 functional groups or if the pin made contact with standing dead vegetation. If the pin made no contact with any live or dead vegetation, it was recorded as “bare soil”. For each subplot (*N* = 40 pins per subplot), we calculated the percent cover of each functional group as the percentage of pins making contact with that functional group, and the percentage exposure of bare soil as the percentage of pins making no contact with any vegetation. Sampling was conducted in 1999 when the exclusion experiment started, and then repeated in 2003 (i.e., 3 years after exclusion) and again in 2016 (17 years after exclusion).

Given the distinct two‐phase mosaic of vegetation in each plot, we conducted separate analyses of changes over time in bare soil‐dominated versus vegetated patches. Using measurements from 1999, we classified each subplot (i.e., the 1‐m radius or 3.14 m^2^ area surrounding each permanent monitoring point) as a “bare patch” if it contained >50% bare soil exposure in 1999, or “vegetated” if <50% bare soil exposure. In 1999, prior to implementation of the herbivore exclusion treatment, bare soil exposure averaged ~80% on subplots classified as bare patches and ~30% on subplots classified as vegetated patches, reflecting the highly bimodal distribution of vegetation cover when measured at the scale of 1‐m radius subplots (see also Augustine, 2003). We analyzed temporal changes in cover of herbaceous functional groups for initially bare patches and for initially vegetated patches, in both cases using a repeated‐measures linear mixed model that also accounted for the randomized complete block design applied at the whole‐plot level, and the division of whole plots into bare versus vegetated subplots based on their initial condition. Analyses were conducted using SAS v9.4.

### 2.2 Measurements of completely barren patches

Methods described above measured the distribution of bare soil at the scale of an individual pin (~1 mm radius), and at the scale of 1‐m radius subplots, where a bare patch was arbitrarily defined as a subplot with >50% of the pins not contacting any vegetation. This definition was useful for quantifying long‐term changes in bare soil exposure on the permanently marked subplots. However, this definition allows bare “patches” to still contain some internal herbaceous cover. At the completion of the experiment in 2016, we used a second method, based on maps created using a high‐resolution Global Positioning System (GPS) recording device, to more precisely quantify the amount and distribution of patches within each whole plot that were nearly completely devoid of herbaceous vegetation cover. Hereafter, we refer to patches mapped by this method as “completely barren patches” to distinguish them from the method used to measure changes in “bare patches” described previously.

We defined a “completely barren patch” as any polygon 1 m^2^ or larger, within which the placement of a 1 m^2^ square quadrat anywhere within the boundary of the polygon would encompass an area containing <2% herbaceous foliar cover, as visually estimated by the mapper. Completely barren patches that were 1–1.5 m^2^ in size were mapped as a single point, which was later buffered with a 0.5 m^2^ radius circle for mapping purposes. Patches larger than 1.5 m^2^ were mapped as polygons using a Trimble GeoXT 3000 (Trimble Companies), set for a maximum dilution of precision of 6.0. During mapping, polygon vertices were each recorded at approximately 1‐m intervals, with ≥10 sets of coordinates recorded and averaged to calculate each vertex coordinate. We use these data to present maps and summary statistics of completely barren patches within each control and exclosure plot.

## 3 RESULTS

### 3.1 Initial conditions

At the start of the experiment, paired whole plots at the three study sites contained a similar percentage of bare versus vegetated patches, with 42% versus 47%, 64% versus 67%, and 72% versus 75% of subplots classified as bare patches in exclosures versus controls, respectively. Within bare patches, bare soil exposure in 1999 was 77 ± 8% inside exclosures versus 80 ± 10% in control plots, respectively (mean + 1 *SE* across sites; contrast for pretreatment means: *p* = .72). The limited herbaceous cover that did occur within bare patches consisted primarily of the LFVG functional group (32%–33% of relative cover in exclosures and controls in 1999; Appendix A). Within vegetated patches, bare soil exposure in 1999 averaged 30 ± 2% in exclosures and 29 ± 5% in controls, respectively (contrast for pretreatment means: *p* = .87). Vegetated patches were dominated by stoloniferous grasses (31% and 27% of relative cover in pretreatment controls and exclosures, respectively), with lesser and relatively even amounts of cover by the remaining 5 functional groups (Appendix A).

### 3.2 Responses to herbivore exclusion

**Figure 2:**
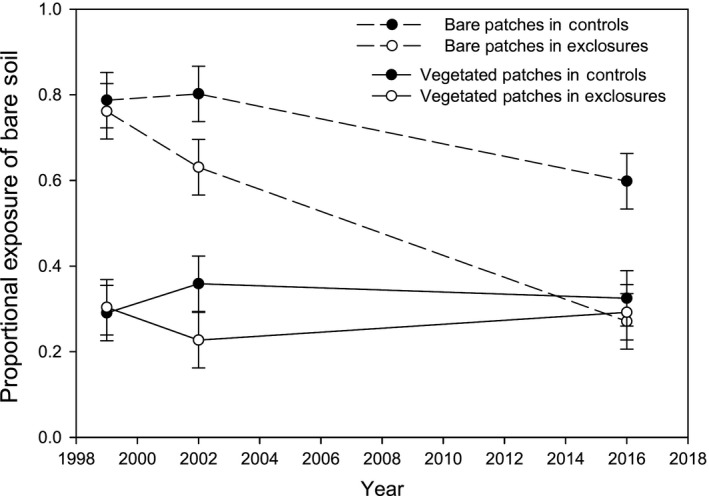
Changes over time in the amount of bare soil within a two‐phase vegetation mosaic consisting of bare patches (1‐m radius plots containing >50% bare soil exposure in 1999) interspersed with vegetated patches (1‐m radius plots containing ≤50% bare soil exposure in 1999) in the presence and absence of large mammalian herbivores (controls vs. exclosures, respectively) at the Mpala Conservancy in Laikipia County, Kenya

Bare soil exposure in bare patches declined more rapidly in exclosures relative to controls (Figure 2). The effect of herbivore removal was marginally evident after 3 years (Treatment contrast for bare patches in 2002: *F*_1,20_ = 3.99, *p* = .059), and highly significant after 17 years (Treatment contrast for bare patches in 2016: *F*_1,20_ = 14.60, *p* = .001). The annual rate of decline in bare soil for initially bare patches inside exclosures was relatively consistent in years 1–3 versus 4–17 (4.2% and 2.9% per year, respectively; Figure 2). In grazed controls, bare soil exposure in bare patches remained constant over the first 3 years and declined over the 17‐year period, but at a significantly lower rate than inside exclosures (1.2% per year; Figure 2). In contrast to the dramatic long‐term effect of herbivore removal on bare patches, we found that in vegetated patches, bare soil exposure remained low and unaffected by herbivore removal both after 3 years (Treatment contrast for vegetated patches in 2002: *F*_1,20_ = 2.36, *p* = .14) and 17 years (Treatment contrast for vegetated patches in 2016: *F*_1,20_ = 0.15, *p* = .71; Figure 2). Furthermore, after 17 years, bare soil exposure within initially bare patches inside exclosures converged with bare soil exposure in vegetated patches both inside and outside exclosures (~29%), and only remained elevated within bare patches in the grazed controls (60%; Figure 2).

**Figure 3:**
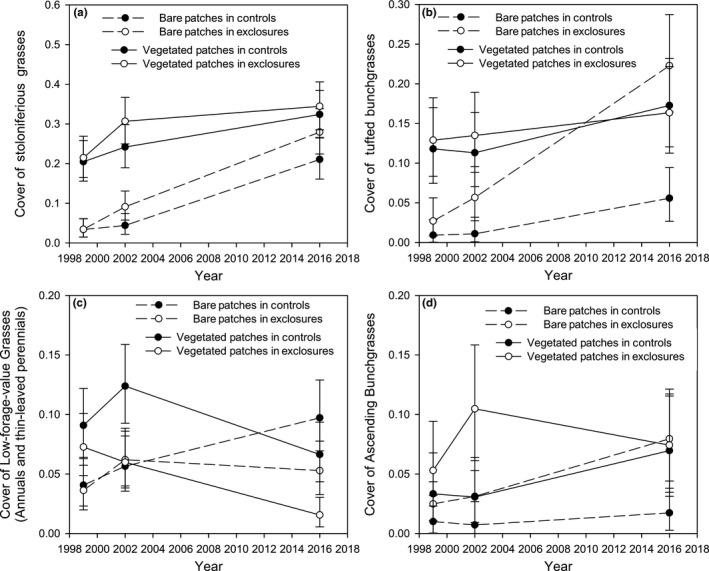
Changes in proportional cover of stoloniferous grasses (a), tufted bunchgrasses (b), low‐forage‐value grasses (c), and ascending bunchgrasses (d) over time within a two‐phase vegetation mosaic consisting of bare patches (1‐m radius plots containing >50% bare soil exposure in 1999) interspersed with vegetated patches (1‐m radius plots containing ≤50% bare soil exposure in 1999) in the presence versus absence of large mammalian herbivores (controls vs. exclosures respectively) at the Mpala Conservancy in Laikipia County, Kenya. See methods for a description of the grass species comprising each functional group. The stoloniferous grasses, tufted bunchgrasses, and ascending bunchgrasses all consisted of species that are commonly grazed by ungulates, whereas the lowforage‐value grasses consisted of annuals and perennials with thin, coarse leaves of low palatability for ungulate grazers

We originally hypothesized that recolonization of bare patches by herbaceous vegetation following herbivore removal would primarily occur by stoloniferous grasses. We found that STG cover increased over time throughout the experiment (Year: *F*_2,20_ = 16.68, *p* < .001), but that herbivore exclusion did not influence the rate of increase either in bare patches (Treatment contrast for bare patches in 2002 and 2016: *F*_1,20_ ≤ 1.43, *p* ≥ .25) or in vegetated patches (Treatment contrast for vegetated patches in 2002 and 2016: *F*_1,20_ ≤ 0.85, *p* ≥ .37; Figure 3a).

Tufted bunchgrasses responded positively to herbivore removal, but this response was contingent on patch type. TBG cover was low in bare patches at the start of the experiment (1%–3%; Figure 3b) and in the presence of herbivores, remained low after 3 years (1%) and 17 years (6%; Figure 3b). Following herbivore removal, TBG cover increased only slightly in the short term (to 7% in 2002; Treatment contrast for bare patches: *F*_1,20_ = 0.90, *p* = .35) but increased dramatically to 24% in 2016 (Treatment contrast for bare patches: *F*_1,20_ = 11.01, *p* = .003). In contrast, TBG cover was unaffected by herbivore removal in vegetated patches, which averaged 13% cover in 1999 and 17% cover in 2016 (Treatment contrast for vegetated patches: *F*_1,20_ = 0.03, *p* = .86).

Ascending bunchgrasses showed similar trends in relation to patch type and herbivore treatment as the tufted bunchgrasses, but patterns were more variable among sites. In bare patches, we detected a marginally significant effect of herbivore removal on ABG cover after 17 years (Treatment contrast in bare patches for 2016: *F*_1,20_ = 3.79, *p* = .066). In vegetated patches, ABG cover averaged 4% in 1999 and 7% in 2016 and was unaffected by herbivore removal (Treatment contrast for vegetated patches in 2016: *F*_1,20_ = 0.39, *p* = .54).

Low‐forage‐value graminoids were unique in being the only functional group for which herbivore removal significantly affected cover in vegetated but not bare patches. Within initially vegetated patches, LFVG cover declined over time in the absence relative to the presence of herbivores (Treatment contrast in 2002: *F*_1,20_ = 4.20, *p* = .053, and in 2016: *F*_1,20_ = 6.11, *p* = .022). In bare patches, LFVG cover was not influenced by herbivore removal (Treatment contrast in 2016: *F*_1,20_ = 2.4, *p* = .14).

The two nongraminoid functional groups (forbs and dwarf shrubs) varied in abundance among years and between patch types (with greater cover in 2002 and in vegetated patches), but neither were affected by herbivore removal (*p* > .15 for treatment contrasts in both patch types in 2002 and 2016; data not shown).

**Figure 4:**
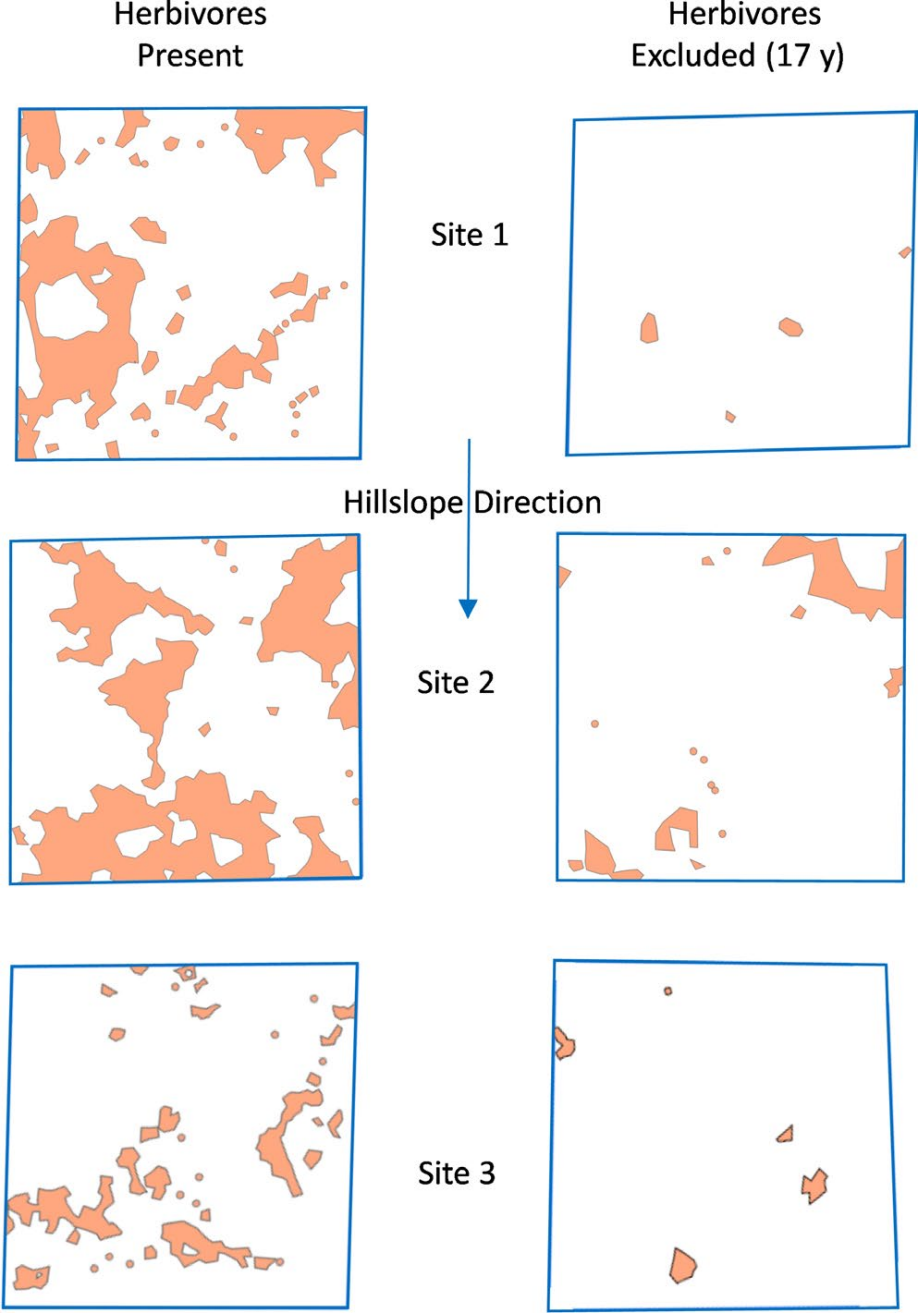
Maps of completely barren patches (see Methods for definition) in the presence of large mammalian herbivores, and in paired sites where large herbivores were absent for the past 17 years, at the Mpala Conservancy in Laikipia County, Kenya in 2016

By 2016, the extent of completely barren patches differed dramatically between exclosures and controls. In the absence of large herbivores, completely barren patches comprised an average of only 3.1% of total plot area (0.7%, 7.3%, and 1.3% at sites 1–3, respectively; Figure 4). In the presence of herbivores, completely barren patches averaged 23.4% of total plot area (24.6%, 35.2%, and 10.5% at sites 1–3, respectively; Figure 4), which was significantly greater than inside exclosures (paired *t* test, *t*_2_ = 4.91, *p* = .039). In the presence of herbivores, most patches were spatially stable over time, with bare patches remaining bare and vegetated patches remaining vegetated (Table 1). Completely, barren patches within exclosures were primarily 1.0–10 m^2^ in size, with a single large patch of 110 m^2^ at one replicate (Figure 4). In contrast, grazed sites contained completely barren patches on the order of 40–100 m^2^ in addition to smaller patches of 1.0–10 m^2^, and the largest mapped barren patch in the grazed plots was 365 m^2^ (Figure 4).

**Table 1:** The percent of all subplots in a given treatment (out of 108) that experienced a given transition between bare and/or vegetated state between 1999 and 2016, in a semi‐arid savanna in Laikipia County, Kenya

Transition type	Treatment	
	Exclosures	Grazed controls
Bare ‐> Bare	8	39
Bare ‐> Vegetated	49	24
Vegetated ‐> Bare	9	10
Vegetated ‐> Vegetated	33	27

## 4 DISCUSSION

Discontinuous vegetation cover, often characterized by densely vegetated patches alternating with nonvegetated patches that are tens to hundreds of meters in diameter, characterizes many arid and semiarid ecosystems worldwide (Aguiar & Sala, 1999; Deblauwe et al., 2008). These two‐phase vegetation mosaics can arise via spatial feedbacks between vegetation cover and surface run‐on/run‐off dynamics, as demonstrated via multiple modeling approaches (e.g., Hillerislambers, Rietkerk, Bosch, Prins, & Kroon, 2001; King & Franz, 2016; Klausmeier, 1999). While ecologists have also theorized that grazing by large herbivores contributes to vegetation pattern formation (Van der Koppel et al., 2002), and that spatial patterns may provide an indicator of grazing‐induced ecosystem degradation (e.g., Kefi et al., 2007), empirical support for this idea has been limited.

Here, we have clearly demonstrated that the removal of large herbivores from a semi‐arid savanna allowed bare soil‐dominated patches to become revegetated, resulting in a far more homogenous distribution of herbaceous vegetation cover compared to the grazed savanna. This shift from a two‐phase mosaic to a continuous herbaceous layer occurred only with long‐term herbivore removal, on the order of 1–2 decades. Although we cannot precisely determine the temporal scale of this shift due to a lack of annual vegetation monitoring, we did find that short‐term removal of herbivores for 1–3 years (Augustine & McNaughton, 2006 and this study) was insufficient to allow bare patches to become revegetated. This result indicates that simply resting a portion of this savanna landscape from livestock grazing for one or two growing seasons will be insufficient to restore bare patches. Rather, much longer‐term reductions in grazing pressure would be necessary, in the absence of other rangeland restoration inputs, to restore bare patches to vegetation cover.

Our study also provides important insights to the concept of alternative stable states in rangeland ecosystems. Bare patches often develop physical surface crusts that reduce water infiltration and increase surface runoff (Franz et al., 2012; Valentin, d'Herbes, & Poesen, 1999). These processes are thought to generate positive feedbacks that prevent vegetation reestablishment and thus could potentially result in an alternative stable state that cannot be reversed via a reduction in grazing pressure alone (Van de Koppel et al., 2002, 1997). Our results show that in the short term, bare patches are indeed relatively stable even in the absence of grazing. However, they do not represent alternative stable states because long‐term removal of herbivores results in grass reestablishment, even in the absence of other restoration treatments, such as creating physical barriers to runoff or raking the soil surface (e.g., Kimiti, Riginos, & Belnap, 2017; Kinyua et al., 2010). At the same time, the ability to substantially reduce or remove large grazers from portions of these landscapes for a decade or more is untenable for pastoralists that rely almost exclusively on livestock to make a living, such that low‐cost methods to restore degraded patches in the presence of grazers are important for the management of these rangelands (Kimiti, Riginos, et al., 2017; Kinyua et al., 2010).

We also note that our findings regarding the temporal scale of vegetation response to grazing removal may be related to the facts that (a) our study system receives a mean annual rainfall >500 mm, and (b) soils have the capacity to form a sealed surface which reduces rainfall infiltration. In some more arid systems characterized by highly variable/unpredictable rainfall (and hence where annual grasses dominate production), as well as in systems where soils have greater infiltration capacity, shorter‐term rest from livestock grazing (e.g., on the order of 1–5 years) may be sufficient to significantly reduce bare soil exposure and enhance forage production (Oba et al., 2000). The size of bare patches may also be an important factor to consider relative to the rate response to herbivore removal. Bare patches at the start of our study were frequently 5–15 m in diameter (Augustine, 2003), similar in size and distribution to those mapped in grazed plots in 2016 (Figure 4). Landscapes with larger bare patches (and correspondingly smaller and less connected vegetated patches) could likely require even longer recovery times than documented in our study (Bestelmeyer, Duniway, James, Burkett, & Havstad, 2013).

Bare patches were relatively stable in location over the course of our study (Table 1), thus raising the question of what factors may have driven their initial spatial distribution. In some cases, the distribution and stability of bare patches in drylands has been show to be influenced by soil properties (e.g., Bestelmeyer, Ward, & Havstad, 2006). Analyses of spatial variation in vegetation cover and soil texture showed that bare and vegetated patches do not differ in the texture of surface soils (0–15 cm; Augustine, 2003). We did not measure soil depth across the plots, but given the small size (50 × 50 m plots), we also suggest that depth to bedrock is not a major factor. While we cannot rule out soil properties as a factor, we speculate that local spatial variation in deterrents to grazing, such as downed, tangled branches of thorn scrub left behind by browsing elephants (Pringle, 2008), clusters of unpalatable herbaceous species, and clusters of spiny shrub saplings (Augustine, 2003) may be important through their interaction with surface run‐off and run‐on patterns (King et al., 2016) to influence the location of vegetated patches.

**Figure 5:**
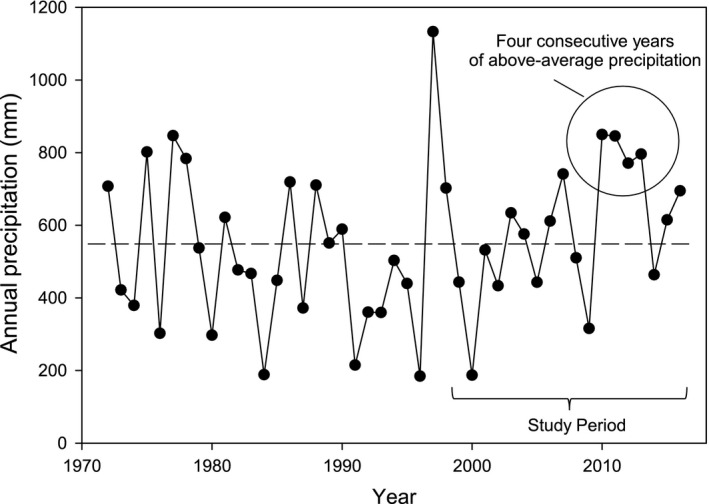
Annual precipitation recorded at a gauge near the center of the study area at the Mpala Conservancy in Laikipia County, Kenya, during 1972–2016. The dashed line shows long‐term mean annual precipitation (546 mm). Note the four consecutive years of above‐average precipitation that occurred during 2010–2013

We originally hypothesized that stoloniferous grasses would be critical in reestablishing vegetative cover in bare patches in the absence of herbivores. However, we found that over the past 17 years, stoloniferous grass cover increased substantially in bare patches in both treatments, reaching a mean of 28% cover in the absence and 21% cover in the presence of herbivores. The finding that stoloniferous grasses were the only functional group to increase significantly over time in the presence of grazers (Figure 3) is consistent with the high grazing tolerance of these species, associated with their prostrate growth form and lack of reliance on reproduction from seed. Additionally, we suggest that the substantial increase over time in both the presence and absence of herbivores may be related to periods of unusually favorable rainfall during our study. Long‐term mean annual precipitation (1972–2016) measured at a gauge near the center of our study area was 546 mm. During the interval from 2003 to 2016, when stoloniferous grasses increased most rapidly in grazed bare patches (Figure 3a), annual precipitation averaged 633 mm. Furthermore, a sequence of four consecutive wet years occurred during 2010–2013, during which time annual precipitation averaged 816 mm (Figure 5). This increase in moisture inputs over four consecutive years was likely an important factor allowing the most grazing‐tolerant plant functional group to increase substantially in bare patches in both grazing treatments.

The functional groups that responded significantly to herbivore removal were the tufted bunchgrasses, which increased in bare patches inside but not outside exclosures, and the low‐forage‐value graminoids, which declined in vegetated patches inside but not outside exclosures. The two most abundant species in the tufted bunchgrass functional group, *E. macrostachyus* and *T. triandra*, are particularly sensitive to grazing because they exhibit minimal inflorescence production where they are accessible to grazers. However, these species can dramatically increase inflorescence production in response to 1 year of protection from grazing (Snyman, Ingram, & Kirkman, 2013). Given that we detected long‐term (17 years) but not short‐term (3‐year) increases in tufted bunchgrass cover in bare patches inside exclosures, we suggest that tufted bunchgrasses require multiple years of protection from grazers in order to establish via seedlings and grow successfully in bare patches, potentially facilitated by the establishment of stoloniferous grasses that helped to reduce water loss to runoff.

The only grazing‐induced shift in functional group composition that we detected within established vegetation patches was a decline in LFVG species inside exclosures relative to grazed plots. We suggest that where grasses were already established prior to herbivore removal, the lack of grazing led to increased competition among grasses and an associated decline in LFVG species. In the absence of grazing, tufted and ascending bunchgrasses as well as the stoloniferous grasses can develop vertically oriented stems and leaf canopies that are taller than LFVG species. Overall, our results indicate that grazers both alter the balance between bare and vegetated patches, and affect the types of grasses dominating both patch types in this semiarid savanna. Thus, large herbivores fundamentally shaped the composition and spatial pattern of the herbaceous layer by maintaining a two‐phase herbaceous mosaic, but bare patches within this mosaic can recover given herbivore removal over sufficiently long time scales, and hence do not represent an alternative stable ecosystem state.

## CONFLICT OF INTEREST

None declared.

## AUTHOR CONTRIBUTIONS

D.A. conceived the experiment and designed methodology; M.S., J.R., and D.A. contributed to long‐term implementation of the experimental design. D.A., J.R., M.S., S.K., B.W., and M.N. all contributed to field data collection and data analyses. D.A. led the writing of the manuscript, and all authors contributed critically to the drafts and gave final approval for publication.

## Data Availability

Data used in this paper are archived at Dryad Digital Repository (https://doi.org/10.5061/dryad.zgmsbcc62).

## APPENDIX A

Relative proportional cover of six plant functional groups inside and outside large herbivore exclosures constructed in 1999 at each of three study sites at the Mpala Ranch and Research Center, Laikipia County, Kenya. STG, stoloniferous grasses; TBG, tufted bunchgrasses; ABG, ascending bunchgrasses; LFVG, low‐forage‐value graminoids; DWS, dwarf shrubs; and FORB, forbs.

**Table:**

Year	Site	Control						Exclosure					
		STG	TBG	ABG	LFVG	DWS	FORB	STG	TBG	ABG	LFVG	DWS	FORB
Relative proportional cover of functional groups in bare patches													
1999	Mean	0.19	0.05	0.04	0.33	0.22	0.18	0.14	0.10	0.11	0.32	0.20	0.14
1999	Site 3	0.31	0.02	0.03	0.31	0.20	0.12	0.16	0.18	0.17	0.11	0.24	0.14
1999	Site 2	0.11	0.06	0.06	0.39	0.09	0.28	0.16	0.09	0.02	0.51	0.03	0.19
1999	Site 1	0.15	0.07	0.03	0.30	0.33	0.13	0.11	0.06	0.14	0.28	0.33	0.08
2002	Mean	0.14	0.06	0.04	0.33	0.05	0.38	0.18	0.10	0.05	0.25	0.17	0.25
2002	Site 3	0.11	0.01	0.05	0.34	0.02	0.46	0.11	0.20	0.11	0.17	0.20	0.22
2002	Site 2	0.18	0.07	0.06	0.30	0.00	0.39	0.28	0.03	0.00	0.31	0.00	0.38
2002	Site 1	0.15	0.13	0.00	0.36	0.14	0.22	0.14	0.09	0.06	0.24	0.31	0.16
2016	Mean	0.37	0.12	0.02	0.28	0.14	0.07	0.27	0.37	0.09	0.06	0.13	0.08
2016	Site 3	0.38	0.05	0.07	0.25	0.19	0.07	0.43	0.02	0.28	0.16	0.09	0.01
2016	Site 2	0.35	0.11	0.02	0.35	0.11	0.07	0.29	0.40	0.01	0.02	0.15	0.13
2016	Site 1	0.39	0.17	0.00	0.23	0.14	0.07	0.15	0.54	0.06	0.05	0.13	0.07
Relative proportional cover of functional groups in vegetated patches													
1999	Mean	0.31	0.18	0.05	0.14	0.17	0.15	0.28	0.16	0.11	0.13	0.19	0.12
1999	Site 3	0.39	0.11	0.07	0.16	0.18	0.09	0.27	0.09	0.18	0.14	0.22	0.10
1999	Site 2	0.28	0.14	0.01	0.21	0.06	0.30	0.32	0.29	0.10	0.15	0.05	0.09
1999	Site 1	0.20	0.32	0.04	0.06	0.25	0.13	0.27	0.18	0.02	0.10	0.24	0.19
2002	Mean	0.31	0.11	0.06	0.18	0.12	0.22	0.26	0.13	0.12	0.10	0.12	0.27
2002	Site 3	0.29	0.06	0.08	0.23	0.07	0.27	0.21	0.08	0.16	0.12	0.13	0.30
2002	Site 2	0.45	0.09	0.01	0.14	0.06	0.26	0.32	0.23	0.11	0.08	0.06	0.20
2002	Site 1	0.26	0.22	0.06	0.11	0.24	0.11	0.32	0.15	0.04	0.07	0.16	0.27
2016	Mean	0.38	0.17	0.12	0.12	0.14	0.07	0.33	0.20	0.19	0.02	0.18	0.07
2016	Site 3	0.41	0.10	0.16	0.16	0.11	0.06	0.35	0.14	0.33	0.03	0.13	0.02
2016	Site 2	0.57	0.13	0.02	0.08	0.12	0.09	0.35	0.19	0.00	0.01	0.30	0.15
2016	Site 1	0.18	0.33	0.12	0.08	0.20	0.08	0.28	0.31	0.09	0.02	0.19	0.12
